# Development of split-force-controlled body weight support (SF-BWS) robot for gait rehabilitation

**DOI:** 10.3389/fnhum.2023.1197380

**Published:** 2023-07-11

**Authors:** Asuka Takai, Tatsuya Teramae, Tomoyuki Noda, Koji Ishihara, Jun-ichiro Furukawa, Hiroaki Fujimoto, Megumi Hatakenaka, Nobukazu Fujita, Akihiro Jino, Yuichi Hiramatsu, Ichiro Miyai, Jun Morimoto

**Affiliations:** ^1^Department of Brain Robot Interface, Brain Information Communication Research Laboratory Group, Advanced Telecommunications Research Institute International (ATR), Kyoto, Japan; ^2^Graduate School of Engineering Division of Mechanical Engineering, Osaka Metropolitan University, Osaka, Japan; ^3^Man-Machine Collaboration Research Team, Guardian Robot Project, RIKEN, Kyoto, Japan; ^4^Neurorehabilitation Research Institute, Morinomiya Hospital, Osaka, Japan; ^5^Graduate School of Informatics, Kyoto University, Kyoto, Japan

**Keywords:** body weight support, pneumatic artificial muscle, force control, center of pressure, body weight shift

## Abstract

This study introduces a body-weight-support (BWS) robot actuated by two pneumatic artificial muscles (PAMs). Conventional BWS devices typically use springs or a single actuator, whereas our robot has a split force-controlled BWS (SF-BWS), in which two force-controlled actuators independently support the left and right sides of the user’s body. To reduce the experience of weight, vertical unweighting support forces are transferred directly to the user’s left and right hips through a newly designed harness with an open space around the shoulder and upper chest area to allow freedom of movement. A motion capture evaluation with three healthy participants confirmed that the proposed harness does not impede upper-body motion during laterally identical force-controlled partial BWS walking, which is quantitatively similar to natural walking. To evaluate our SF-BWS robot, we performed a force-tracking and split-force control task using different simulated load weight setups (40, 50, and 60 kg masses). The split-force control task, providing independent force references to each PAM and conducted with a 60 kg mass and a test bench, demonstrates that our SF-BWS robot is capable of shifting human body weight in the mediolateral direction. The SF-BWS robot successfully controlled the two PAMs to generate the desired vertical support forces.

## 1. Introduction

Stroke patients need to be supported on the paralyzed side with a sufficiently large external force to walk safely. Therefore, at clinical sites, gait training is conducted for patients who can walk on treadmills with the aid of a device that lifts the body vertically (Hesse, 2008). Previous clinical studies have reported that the benefits of body weight-supported treadmill training (BWSTT) for post-stroke gait rehabilitation are comparable to those of treadmill training without BWS (Visintin et al., 1998; Mehrholz et al., 2017).

Impaired body orientation results in the deviation of the center of pressure [CoP, a projection of the center of mass (CoM) on the ground] in the mediolateral (left-right) direction and is highly correlated with balance disorders and walking disorders (Dai et al., 2021). Therefore, in addition to the partial body weight support against gravity, a configuration for manipulating the CoP in the mediolateral direction is required for patients with hemiparetic stroke, the main target of this study. In fact, when natural walking is unimpaired, the CoM oscillates smoothly both vertically and mediolaterally, and the pelvic list contributes to the mediolateral displacement of the CoM (Lin et al., 2014). However, conventional BWS devices used at clinical sites lift both sides of the body with identical forces using a solo actuator. Hence, conventional BWS devices cannot intervene in pelvic rotations. In addition, these devices can only support a constant force and cannot dynamically change the amount of support provided during walking.

**FIGURE 1:**
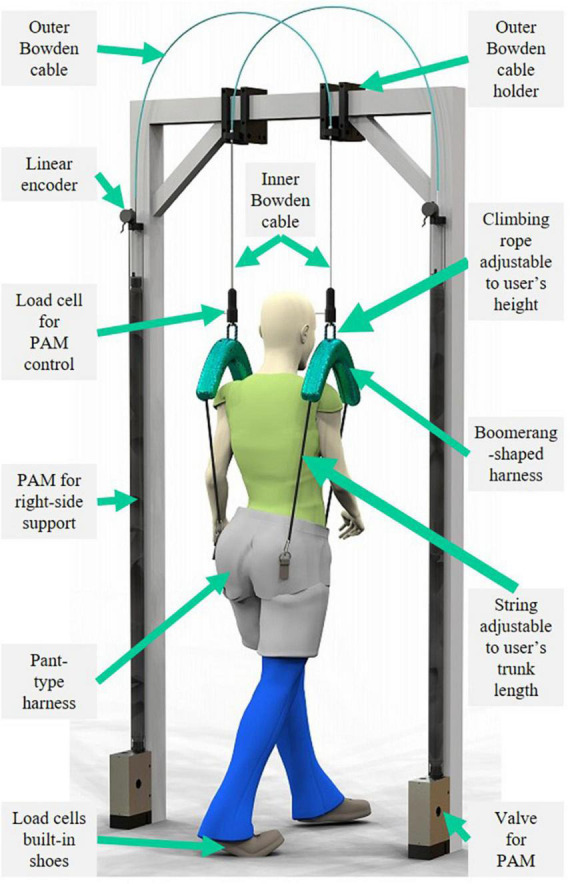
Proposed Split-Force controlled body-weight-support (SF-BWS) robot. SF-BWS independently supports both sides of the human body using paired pneumatic artificial muscles (PAMs). Uniquely designed harnesses with green covers transmit assist forces to the user. Boomerang-shaped parts split force into two bottom ends, and adjustable strings attached at both ends are connected to the pant-type harness.

In this study, we propose a BWS robot with the capability to intervene in pelvic rotation to induce mediolateral CoP modulation by independently applying vertical dynamical forces on each side of the body, called a Split-Force controlled BWS (SF-BWS) robot (see Figure 1). It is conceivable that a BWS system requires compliance to allow for more natural walking and reduce discomfort (Chen et al., 2005). To achieve compliant support, our previous study (Furukawa et al., 2016) proposed an assisting robot that used a pneumatic artificial muscle (PAM) to lift just one side of the body. Because PAMs can deliver large forces and are compliant, they are more suitable for human movement than geared motors. Elastic actuators (Kwak et al., 2017) other than PAM can be adopted to achieve a comparable weight, size, and force amplitude, and PAM presents challenges in modeling and precise control. However, PAM can reduce the number of mechanical parts for elasticity owing to its low intrinsic impedance. Minimal mechanical complexity is advantageous in clinical settings.

**FIGURE 2:**
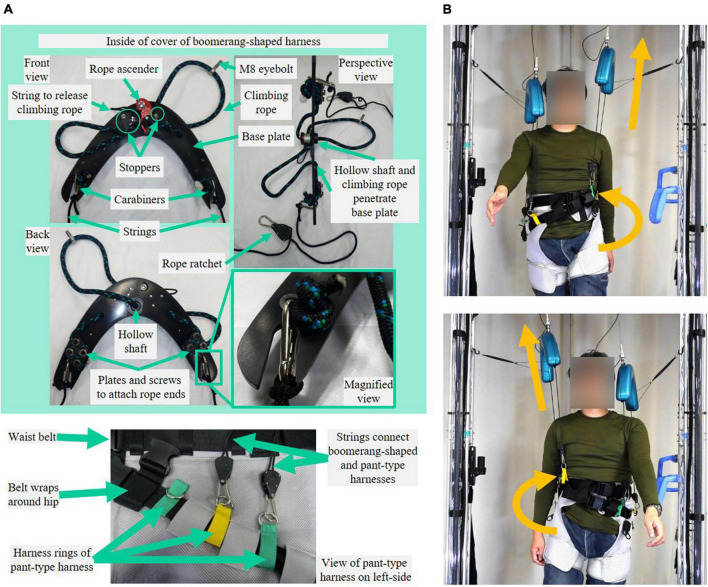
Mechanical design and applicable force of SF-BWS robot. **(A)** Inner Bowden cable [the tip of pneumatic artificial muscles (PAMs)] is attached to climbing rope via load cell, used for the force control. In order to supply force while always tensioning the inner Bowden cable, the vertical position of the uniquely designed harness must be adjusted to the user’s height. The rope ascender inside the cover can adjust the length of the climbing rope. Harness rings of a pant-type harness are located around the trochanter. The rope ratchet allows for adjusting the string length to the user’s trunk length to tension the inner Bowden cable and the string. In order to be worn and removed, the pant-type harness and Boomerang-shaped harness are easily disconnected by parts at the end of the rope ratchet. **(B)** Illustration of independent vertical assist of SF-BWS applied on the user to cause pelvic obliquity.

This study extends the previous robot and introduces a newly designed mechanical harness (Figure 2A) that avoids impeding natural walking patterns. This study compares upper body motion during natural and laterally identical force-controlled partial BWS walking with three healthy participants wearing the proposed harness. Additionally, this study demonstrates that the SF-BWS robot can perform a force-tracking task using a sinusoidal reference with various simulated load weight setups (40, 50, and 60 kg masses). We also demonstrate the split-force control performance of the SF-BWS robot in terms of intervening in the CoP using a 60 kg mass placed on a test bench. The split-force control task is an imitation task of the double stance gait phase in which the CoP is actively moved mediolaterally by pelvic oblique force through the two PAMs. The patient’s weight shift during SF-BWS assisted walking was confirmed by physiotherapists in our previous report. Thus, in this study, we quantitatively demonstrated the force control performance in the ideal state of the double-leg support using a test bench in which the weight is stably supported by both the left and right legs, with no fluctuations in the CoM.

The contributions of this study can be summarized as follows:

(1) A novel body-weight-support (BWS) robot (Figure 1) that can independently assist each side of the user’s body was developed. A uniquely shaped harness and attachments (Figure 2A) with an open space around the shoulder and upper chest area to allow for freedom of movement and to avoid interfering with the user’s natural movements while supporting vertically was developed (Figure 2B).
(2) Long pneumatic artificial muscles (PAMs) with mechanical compliance, aiming for compliant body support in a rehabilitation program, were adopted. The PAMs can generate a sufficiently large force to assist the user’s body. Long-size PAMs were customized using commercially available rubber components to achieve the necessary movement range. The developed BWS robot demonstrated precise control over large forces using our PAM control strategy. Unlike clinically available BWS devices, which use a passive component such as a spring, the support forces can be actively adjusted according to sensor information.
(3) To demonstrate the split-force control performance, a split-force control task experiment was conducted. SF-BWS is a robot that partially supports body weight and also moves the CoP in mediolateral directions while a user is walking. In the double stance phase, the position of the CoP quickly shifts from one leg to the other, while the position changes little in the single stance phase (Hof et al., 2007). A test bench was built to evaluate the performance of CoP modulation in the double stance phase. The force controller of SF-BWS tracks a force reference to accomplish a sinusoidal CoP reference trajectory.

## 2. Related works

### 2.1. Mechanical features of BWS robots

Previous studies have introduced BWS robots with different mechanical design concepts. Many BWS robots actuate the suspension mechanism and allow users to move around a floor, lifting both sides of the body using a solo actuator (Hidler et al., 2011; Vallery et al., 2013; Mignardot et al., 2017; Plooij et al., 2018). These robots provide overground walking rehabilitation, which is fundamentally different from treadmill walking; however, they have not been developed for modulating impaired body orientation. Fewer BWS robots provide dynamic and asymmetric support for each side of the body using more than two actuators. Kwak et al. (2017) developed a robot with a pair of compact series elastic actuators (SEA) that assist the left and right sides of the body to provide vertical forces while freeing the user’s natural medial-lateral movement. Van Thuc and Yamamoto (2016) designed a BWS robot with four pneumatic actuators arranged at the corners of the walking space to follow the displacement of the user’s CoP and adopt the virtual application point of support. Dong et al. (2021) developed a mobile-type BWS walker with two variable stiffness actuators on both sides of the lower limbs to relieve part of the body weight. Meanwhile, robots intended to actuate the pelvis have also been investigated. Pietrusinski et al. (2010) developed a robot that generates a force field around the pelvis using linear electromagnetic actuators placed on the left and right sides of the user’s body to guide pelvic obliquity. Aoyagi et al. (2007) designed a pelvis assist manipulator with pneumatic cylinders to assist the pelvic motion that can be used together with a separate overhead BWS system. Khan et al. (2018) designed a trainer for static and dynamic CoM stability. Patton et al. (2008) developed a mobile base system that holds the torso using a pelvic belt to provide partial BWS and postural control on the torso. Luu et al. (2014) designed a pelvic assistance mechanism holding the pelvis by a rigid frame to provide BWS and body weight shifting during gait rehabilitation. The advantages and limitations of past studies are discussed in the following section.

### 2.2. Harness design

Even though the point of contact between a human and a BWS robot drastically affects walking patterns, it has rarely been studied (Plooij et al., 2021). Most BWS robots use a harness that shares points of pressure with the lumbar-thoracic harness and thigh straps (Hidler et al., 2011; Kwak et al., 2017; Mignardot et al., 2017). While conventional harnesses wrap around the upper torso and connect double shoulder points to a spreader bar, Van Thuc and Yamamoto (2016) designed an original lumbar-thoracic harness with four shoulder point connections to four PAMs. The trunk is stabilized if the attachment points are as high as those at the shoulder or trunk level (Plooij et al., 2021). Meanwhile, a pelvic brace (Aoyagi et al., 2007; Pietrusinski et al., 2010), pelvic belt (Patton et al., 2008; Khan et al., 2018), and pelvic frame (Luu et al., 2014) are selectively used to apply the desired force around the pelvis. Attachments on the sides of the pelvis are beneficial in that they do not impede the forward-leaning of the upper body to take a step, compared to assists at a higher position (Plooij et al., 2021). However, Plooij et al. (2021) argued that the mechanical structure inhibits pelvic rotations along the vertical axis. Responding to these suggestions, we aim to design a harness and attachments to intervene in the pelvic obliquity, but not to impede natural walking movement, introduced in Section “3. Mechanical design” and evaluated in Sections “5.1. Mechanical design evaluation and 6.1. Mechanical design evaluation.”

### 2.3. Support force range

Previous studies have demonstrated that the support force of the existing BWS systems is weak in modulating spatiotemporal walking patterns. For example, Dragunas and Gordon (2016) reported that the step width and step length were not significantly modulated by BWS devices with less than 60% of the participant weight in their experiment with healthy participants. Similarly, Tran et al. (2020) reported that abnormal stride length was not corrected by counterweight supports with less than 70% of the weight of healthy participants who reproduced the gait pattern of hemiplegia. From this perspective, the range of force control of Pietrusinski’s robot (Pietrusinski et al., 2010), which is less than 10 N, is insufficient for modulation. Other BWS robots can provide large forces but have evaluated the force tracking performance in a relatively smaller range. For example, a robot by Dong et al. (2021) can provide more than 100% of body weight but test with less than 20%. A robot by Kwak et al. (2017) can provide 300 N at maximum but evaluate the robot with a small range using a 40–80 N sinusoidal force reference. Only Van Thuc and Yamamoto (2016) showed their robot capability with 500 N at maximum and a 100–500 N sinusoidal reference tracking performance at various frequencies. We refer to this study to conduct a force-tracking task in Sections “5.2. Force tracking task with mass and 6.2. Force tracking task with mass.”

### 2.4. Evaluation approaches to CoM/CoP modulation

Conventional BWS devices relieve the load on therapists to support and provide stability to the patients performing gait training. However, many manual assists are still required, such as encouraging weight shift with pelvic motion (Aoyagi et al., 2007). Thus, pelvic actuation and unconstrained natural movements have been investigated. Ichinose et al. (2003) recorded and replayed a pelvic motion and showed pelvic position tracking performance of position/force control during unimpaired human walking. Aoyagi et al. (2007) further developed synchronization control for the robot and demonstrated its position-tracking performance with individuals with spinal cord injury. Pietrusinski et al. (2014) showed pelvic obliquity tracking performance with healthy participants where the reference was measured from a healthy participant. Vashista et al. (2016) showed pelvic anterior-posterior and vertical motion-tracking performance. Dong et al. (2021) demonstrated mediolateral pelvic trajectory tracking performance. Therefore, although previous robots have the ability to control the CoM/CoP through pelvic control, the actual position controllability of the CoM/CoP has not been well discussed. To date, only two studies have investigated weight-shifting performance. Kwak et al. (2022) have shown tracking performance in the vertical displacement of a hanged mass CoM. Van Thuc and Yamamoto (2016) showed CoP tracking performance with three healthy participants. Moreover, they were designed to track the CoP measured with load cells so that the right-side actuator increases the force when the CoP moves to the right foot. However, when a robot refers to the abnormal pattern of a patient’s gait, it is impossible to modulate their impaired gait. In the clinical setting, a therapist gently pushes the patient’s pelvis and transfers the body weight to the stance leg (Yagura et al., 2006). Thus, in this study, the robot aimed to lead the users in shifting their body weight. To prove the concept of this function, we evaluate the robot control performance in a split-force control task using a test bench in Sections “5.3. Split-force control task with test bench and 6.3. Split-force control task with test bench.”

## 3. Mechanical design

Figure 1 shows the newly designed SF-BWS robot. Each half of the human body was lifted using a separate nested cylinder PAM (NcPAM). The mechanical designs of the NcPAMs were described in detail in our previous papers on the lower (Noda et al., 2018a) and upper extremity exoskeletons (Noda et al., 2018b). The PAM’s contract force is transmitted through the inner Bowden cable, without losing the tension, by a spring built into the PAM placed in a nested cylinder. The pair of PAMs in Polycarbonate tubes are equipped with holders for the columns of a conventional BWS device (PneuWeight, Pneumex Inc.). We used this device’s columns but did not utilize the BWS function. In other words, our robot can be attached to a conventional BWS device installed in a clinic. Note that SF-BWS is not intended for use together with the conventional BWS device.

The inner Bowden cable emerged from the outer Bowden cable holder attached to the center of the top beam to provide vertical support while walking on the treadmill. In this experiment, the left and right inner cables were arranged at intervals of 365 mm, which is the sum of the average distances of the greater trochanters of Japanese men of average height (298.4 mm), with an offset of approximately 33 mm from each harness cover. To fulfill a long operational range, we customized 1,640 mm long NcPAMs with a contraction rate of 25% from their natural length. To use NcPAMs for the walking training of severely paralyzed patients, a force large enough to support the sufficient weight of the patient is required, as introduced in Section “2. Related works.” Therefore, we selected PAMs with a diameter of 20 mm, which could generate a maximum force of 1,500 N.

The pulling forces were transmitted to the user via uniquely shaped harnesses with green covers (weight: 1.7 kg). To supply a force while always tensioning the inner Bowden cable, the vertical position of the developed harness and the length of the string that connects to the pant-type harness (Moritoh Co., Ltd., Japan) must be adjusted to the height of the user. It can be used by users with heights ranging from 1,450 to 1,800 mm and weights of up to 100 kg. The proposed harness contained a rope ascender to adjust the height of the harness for the user (Figure 2A).

To transmit split forces into the user’s body, a new harness must be designed. We designed our harness to support the user’s left and right hips, with an open space around the shoulder and upper chest area to allow for freedom of movement (Figure 1). The Boomerang-shaped black base plate was cut from a 5 mm thick polyoxymethylene (POM) plate using a commercial laser cutting machine. Commercially available rope ascenders (Kong Duck) were used, which are climbing tools for adjusting climbing ropes with a maximum load of 400 kg. A 13 mm climbing rope was passed through an M8 eyebolt screwed to a load cell. The rope also passes through the hollow shaft that screws to the rope ascender and through the holes in the base plate. This reduced the thickness of the harness and was the only way to attach the rope ascender without creating additional holes or cuts in the rope ascender. Since the rope ascender rotates around the hollow shaft, stoppers are attached next to the rope ascender. Both ends of the rope were tied around four screws and fixed to the base plate with 3 mm polycarbonate plates. Two holes and slits on both edges of the boomerang-shaped base plate were hooked to a carabiner and swung at a certain range. The above mechanical parts are all covered by a 3D printed cover, except for the string of the ascender to release the rope. Therefore, the length of the climbing rope can be adjusted without opening the cover. A string is tied to the carabiner, and its length can be adjusted by the rope ratchet at the other end. The rope ratchet and strings are also commercially available (LEDGLE), and their maximum load is 68 kg each. The proposed and pant-type harnesses were connected at the front and back sides of the pelvis with separate strings. Thus, the point of contact between the robot and the human is on the sides of the pelvis (hips), without a rigid structure. This provides PAM forces directly around the pelvis without constraining natural human movement during walking. Furthermore, we confirmed that our harnesses could be used for patients (Fujimoto et al., 2018).

## 4. Controller design

**FIGURE 3:**
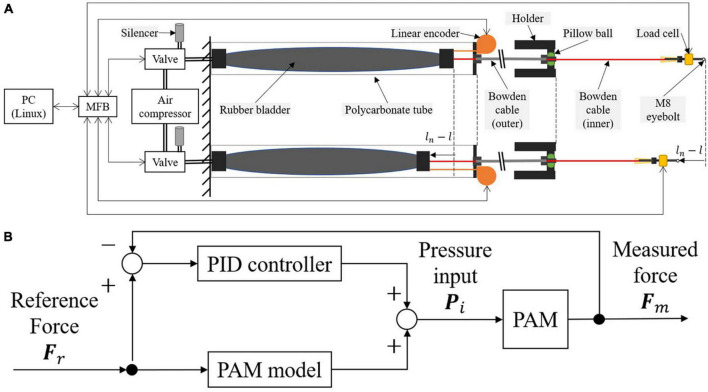
Pneumatic Artificial Muscle (PAM) actuation strategy. **(A)** System schematic of PAM controller. **(B)** Diagram of force controller.

Pneumatic artificial muscle converts pressure to the contraction force by the spiral fiber built into the rubber bladder. We used a 3.7 kW compressor (Hitachi Industrial Equipment Systems Co., Ltd., SRL-A3.7DV). We used a proportional pressure control valve (Norgren Ltd., VP5010SBJ111H00) for each PAM and a multi-function board (MFB) (equipped with AD converters, DA converters, and quadrature encoder interface) connected to the control PC (Linux with Xenomai for real-time feedback loop) via 100 Mbps TCP/IP Ethernet to control the input voltage to the valve (Figure 3A). The PAM contraction force was defined by the input pressure and length. Linear encoders (MTL Inc., MLS-30-450E-1000) were used to measure the PAM length. We evaluated the actual pulling forces by the load cells (UNIPULS Corp., UNCLB-1kN) located at the inner Bowden cable end. The control frequency and sampling framerate of the above sensors were 250 Hz.

The PAM force controller consists of a feedforward PAM model and a proportional integral derivative (PID) feedback (Figure 3B). The feedforward model considers the static characteristic of the PAM, meaning it models the non-linear relationship of variables when the PAM contraction length and applied pressure are near constant, and the external and generated forces are balanced. Meanwhile, PAM dynamics are non-linear and include pneumatic and mechanical dynamics. Specifically, this includes dynamics related to PAM contraction, friction between outer and inner Bowden cables, and pneumatic control valve dynamics. The effect of these dynamics depends on the operating speed of the PAM. Assuming that this effect is sufficiently small, this study compensates for them using PID feedback control.

The PAM model converts the reference force into the required pressure input, and its parameters were calibrated by measuring the PAM contraction rate α, pressure **P**, and force **F**. According to Teramae et al. (2014), the feedforward pressure *P*_*ff*_ at the equilibrium states can be derived as follows:


(1) $$P_{ff}=\frac{\left(F_{r}-F_{l}\right)\,\left(P_{u}-P_{l}\right)}{F_{u}-F_{l}}+P_{l},$$

where *P_u_* and *P_l_* are the constant maximum and minimum pressures set for SF-BWS (0.6 and 0.1 MPa), respectively. Quadratic force models *F_u_* and *F_l_* are the function of the contraction rate α.


(2) $$F_{u}=a_{u}\,\alpha^{2}+b_{u}\,\alpha +c_{u},$$

(3) $$F_{l}=a_{l}\,\alpha^{2}+b_{l}\,\alpha +c_{l},$$

(4) $$\alpha =l/l_{n},$$

where *l* is the current PAM length and *l_n_* is the natural length of PAM. The details on the feedforward PAM model are adopted from Teramae et al. (2014).

We also adopted a feedback controller to cope with the modeling error and applied a correction based on proportional, integral, and derivative terms. The feedback pressure *P*_*fb*_ was designed as follows:


(5) $$P_{f\,b}=K_{p}\,e+K_{i}\,\int edt+K_{d}\,\frac{d}{d\,t}\,e,$$

where *e* = *F*_*r*_ − *F*_*m*_ is the force error, *F_r_* is the reference force, and *F_m_* is the measured force. The proportional gain *K_p_*, integral gain *K_i_*, and derivative gain *K_d_* were individually tuned to compensate for the force error.

Thus, the input pressure *P_i_* required to generate the reference force *F_r_* is derived by:


(6) $$P_{i}\,\left(t\right)=P_{f\,f}\,\left(\text{t}\right)+P_{f\,b}\,\left(t\right)$$

## 5. Experimental setups

### 5.1. Mechanical design evaluation

**FIGURE 4:**
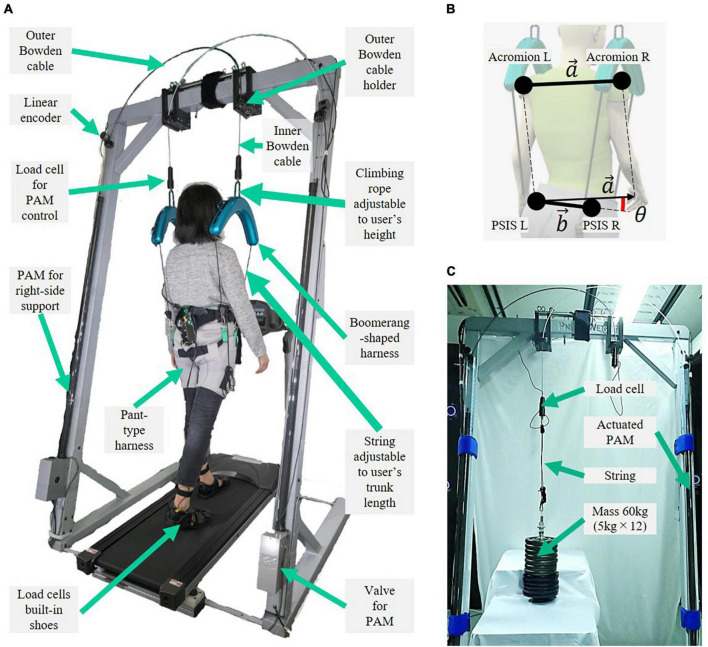
Experimental setup for **(A)** mechanical design evaluation, **(B)** marker positions and three-dimensional angle between two vectors that connect left and right acromie and posterior superior iliac spine (PSIS), and **(C)** force tracking task using a 60 kg mass.

The upper body motions were compared during natural and laterally identical force-controlled 30% BWS walking. Three healthy participants (Participant A: 71-year-old male, Participant B: 71-year-old female, and Participant C: 32-year-old female) wore a motion capture suit (Prime 17W OptiTrack, NaturalPoint Inc.) and walked on a treadmill at their preferred speed for 1 min (Figure 4A). Eight cameras were arranged surrounding the SF-BWS, all at 2.5 m high from the ground. Reflection markers were attached just above the left and right acromie on the motion capture suit. For the left and right posterior superior iliac spine (PSIS), markers were attached to the pant-type harness. We compared the orientation of the upper body using the angle between the vectors passing through these four points (Figure 4B). We designed an analog low-pass filter with a passband within 3 dB from 20/250/2 rad/s while rejecting at least −40 dB below 100/250/2 rad/s (sampling frequency was 250 Hz). The order was 2. Marker positions were passed through the filter before calculating the following.

The three-dimensional angle between the two vectors that connect the left and right acromie $\vec{a}=\left(a_{x},a_{y},a_{z}\right)$ and PSISs $\vec{b}=\left(b_{x},b_{y},b_{z}\right)$ was obtained using the following equations:


(7) $$\theta =\cos^{-1}\frac{a_{x}\,b_{x}+a_{y}\,b_{y}+a_{z}\,b_{z}}{\sqrt{a_{x}^{2}+a_{y}^{2}+a_{z}^{2}}\,\sqrt{b_{x}^{2}+b_{y}^{2}+b_{z}^{2}}}.$$

Here, the straight-line vectors are $\vec{a}=\vec{P_{L\,A}\,P_{R\,A}}$ and $\vec{b}=\vec{P_{L\,P}\,P_{R\,P}}$, where *P*_*LA*_ = [*x*_*LA*_, *y*_*LA*_, *z*_*LA*_], *P*_*RA*_ = [*x*_*RA*_, *y*_*RA*_, *z*_*RA*_], *P*_*LP*_ = [*x*_*LP*_, *y*_*LP*_, *z*_*LP*_], and *P*_*RP*_ = [*x*_*RP*_, *y*_*RP*_, *z*_*RP*_]. The angle time history was separated based on the gait cycles. The step was detected using the foot load cells in Figure 4A built into the pair of shoes (weight: 1.2 kg). Each heel strike was detected if the load cell force exceeded the threshold. In this experiment, the age of the two participants was high, and they had no previous experience with BWS; therefore, safety was prioritized and 30% BWS was set. This is because Hesse (2008) reported that unloading more than 30% BW is not recommended due to decreased muscle activity in the lower extremities. The reference force **F**_*r*_ = [*F*_*rL*_, *F*_*rR*_]^*T*^ in Figure 3 is as follows:


(8) $$F_{r\,L}=F_{r\,R}=\frac{1}{2}\,\beta \,M\,g,$$

where a partial body weight support rate is β, the participant’s weight is *M*, and the gravity acceleration is *g*. All participants provided written informed consent before participation, and the local ethical committee approved the study protocol.

### 5.2. Force tracking task with mass

To evaluate the force controller consisting of the feedback PAM model and PID feedback, we demonstrated the control performance of the wide range of force tracking using the SF-BWS. Figure 4C shows the experimental setup. At this point, the mass and the Bowden cables of the PAM were connected by a string. Since this string was elastic, the PAM contracted even when the mass did not change its height; the nonlinearity of the PAM increases most when it is elastic. Therefore, this task allowed us to evaluate the control performance of the proposed method in a system with PAM nonlinearities.

As it is required to have a large enough force to modulate spatiotemporal walking patterns by previous studies (Dragunas and Gordon, 2016; Tran et al., 2020), we used a mass assuming the human body and hooked it at the end of the inner Bowden cable, as shown in Figure 4C. It can be stabilized by support at one point; thus, the mass is hooked directly without the proposed harness. For assisting patients, the weight of the mass was 60 kg. We set the force tracking task with the reference force with a sinusoidal wave to show that SF-BWS can dynamically change the amount of support in real time based on measured interaction forces. Since the possible amplitude of partial BWS is approximately 30–70% of the body weight according to previous studies (Hesse, 2008; Dragunas and Gordon, 2016; Tran et al., 2020), we set the minimum support at 176.4 N (60 kg × 9.8 m/s^2^ × 30 %). The range of the reference is up to 600 N per PAM to fully compensate for the mass weight; thus, SF-BWS totally supports 1,200 N. Note that we limited the maximum force reference up to 600 N for safety. The PAM itself can generate a much larger force (1,500 N). In this experiment, the right PAM is not used. Thus, the reference force **F**_*r*_ = [*F*_*rL*_, 0]^*T*^ in Figure 3 at time step *t* is as follows:


(9) $$F_{r\,L}=A\,s\,i\,n\,\left(2\,\pi \,f\,t\right)+B,$$

where the amplitude *A* of the sinusoidal reference is 211.8 N, the bias *B* is 388.2 N, and the frequency *f* is 0.5 Hz [referred to Van Thuc and Yamamoto’s (2016) study].

In addition, we performed a force-tracking task using different simulated load weight setups (40, 50, and 60 kg masses). The smallest weight was selected because the 2.5 percentile of elderly Japanese people’s body weight is 40 kg according to a database (Kouchi and Mochimaru, 2005). To examine the 30–70% weight support, the reference force was set as $A=\frac{1}{2}\,\left(0.7-0.3\right)\,M$, *B* = *A* + 0.3*M*, and *f* = 0.5 in Eq. (9). The mass was in contact with the ground, while the air pressure was zero; that is, PAM was at its natural length and the length of the string connecting PAM and the mass was adjusted to be tightened. The string was released and retightened each time we changed the mass. Again, the right PAM was not used in this experiment, so *F*_*rR*_ was set to zero.

We derived that the coefficients *a_u_*, *b_u_*, *c_u_*, *a_l_*, *b_l_*, and *c_l_* are 0.00013, −0.00361, 336.6179, 0.00017, 519.0473, and 38.66615 for left PAM in Eqs. (2) and (3). We set *K_p_*, *K_i_*, and *K_d_* as 6.0 × 10^−2^, 7.0 × 10^−4^, *and* 6.0 × 10^−5^ in Eq. (5).

### 5.3. Split-force control task with test bench

**FIGURE 5:**
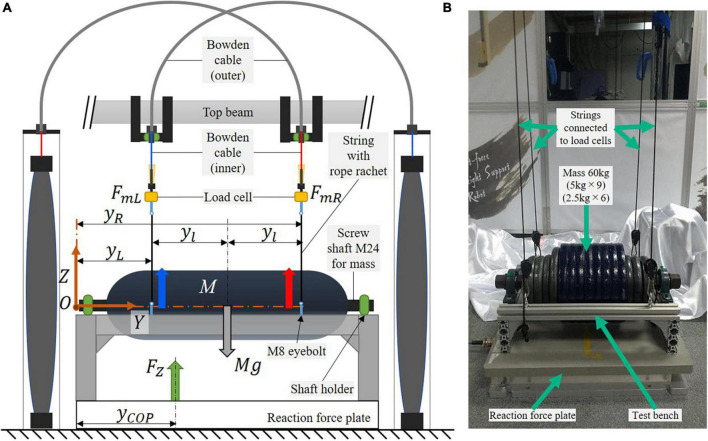
Test bench on reaction force plate, which is used to demonstrate the split-force control performance of SF-BWS. **(A)** Dynamics model. **(B)** Experimental setup.

This section illustrates the force control strategy for the proposed SF-BWS robot in a split-force control task. We use a test bench to demonstrate that SF-BWS can precisely track arbitral desired force. To test the functionality indicated in contribution 3 mentioned in the introduction, the test bench on a reaction force plate (AMTI Inc., BP400600-1000) mimicked the human body. We hooked the bench at the end of the inner Bowden cable via load cells. Figure 5A shows the configuration of the test bench model. Figure 5B shows the bench used in this task. The weight of the mass is 60 kg. The size of the test bench made of aluminum frames is 530 × 300 × 120 (width × depth × height). The masses are threaded through a screw shaft (M24, 606 mm) and screwed to the top of the bench with holders. M8 eyebolts are also screwed on the top of the bench to hook carabiners. A string is tied to the carabiner, and its length is adjustable by the rope ratchet at the other end. The string goes through the M8 eyebolt screwed to the load cell.

The equations for the equilibrium of force and moment around the origin *O* can be described with the following equations:


(10) $$F_{L}+F_{R}+F_{Z}-M\,g=0,$$

(11) $$-F_{L}\,y_{L}-F_{R}\,y_{R}-F_{Z}\,y_{C\,O\,P}+M\,g\,\left(y_{L}+y_{l}\right)=0,$$

where the reaction force measured on the reaction force plate is *F_Z_*. The 60 kg mass is located in the middle of the bench, where the height difference between the mass CoM and the positions where inner Bowden cables are attached to the test bench (weight: 7.55 kg) is negligible. Thus, *M* in Eqs. (10) and (11) is the sum of the weight of the mass and the bench. PAMs are attached in the distance *y_l_* from the bench center. The distance from the origin to the attachment location of the left PAM is defined as *y_L_*, and that of the right PAM is *y_R_*. The physical parameters of the test bench are provided in Table 1. When a partial body weight support rate is β, the partial BWS force of PAMs *F*_*rL*_ and *F*_*rR*_ are


(12) $$F_{r\,L}+F_{r\,R}=\beta \,M\,g,$$

**TABLE 1:** Physical parameters of test bench.

Parameter	Value
*M*	67.55 kg
*y_l_*	0.1825 m
*y_L_*	0.1175 m
*y_R_*	0.4825 m

If we give SF-BWS a desired trajectory of the CoP, the forces are determined geometrically to accomplish the target CoP position. Thus, the pelvic rotational force of PAMs *F*_*rL*_ and *F*_*rR*_ at time step *t* will be


(13) $$F_{r\,R}=\frac{\left(1-\beta \right)\,y_{L}+y_{l}-\left(1-\beta \right)\,y_{C\,O\,P}}{2\,l}\,M\,g,$$

(14) $$F_{r\,L}=\beta \,M\,g-F_{R},$$

where the CoP reference is


(15) $$y_{C\,O\,P}=A\,s\,i\,n\,\left(2\,\pi \,f\,t\right)+B.$$

Since the amplitude of the natural human CoP trajectory is assumed within stride width, which is the distance between the two heels during the double stance, and the normal stride width of adults is between 3 to 8 cm (Wellmon, 2007), the CoP reference was set as a sinusoidal wave with *A* = 0.050 m, *B* = 0.300 m where the center of the reaction force plate locates and *f* = 1.0 Hz for Eq. (15), which was set by referring to the cadence reported in previous studies [52.5 ± 17.0 steps/min by Tudor-Locke et al. (2020) at 0.8 km/h treadmill speed where the upper range walking speed of most stroke patients (Capó-Lugo et al., 2012)]. Then, the required forces for two PAMs were obtained from Eq. (13) and Eq. (14). The left PAM’s coefficients are the same as the force-tracking task (explained above). We derived that the coefficients *a_u_*, *b_u_*, *c_u_*, *a_l_*, *b_l_*, and *c_l_* are −0.00059, −0.00021, −0.00117, −0.00035, −0.00016, and −0.00186 for right PAM in Eqs. (2) and (3). We set the proportional gain *K_p_*, integral gain *K_i_* and derivative gain *K_d_* as 8.0 × 10^−2^, 2.0 × 10^−3^, *and* 5.0 × 10^−3^ for the right-side PAM. For the left-side PAM, *K_p_*, *K_i_*, and *K_d_* are 3.0 × 10^−2^, 1.5 × 10^−3^, *and* 5.0 × 10^−3^, respectively, in Eq. (5). We conducted experiments in two conditions: (1) **First condition:** 1-second single-wave reference for the reproductivity check, and (2) **Second condition:** 20 s block-designed reference.

We used load cells for feedback control. Thus, the measured forces **F**_*m*_ = [*F*_*mL*_, *F*_*mR*_]^*T*^ in Figure 3.

The reaction force *F_Z_* is not always measurable at clinical sites; thus, we did not use it for feedback control. We used the reaction force plate to evaluate the CoP displacement in the Y-direction as follows:


(16) $$y_{C\,O\,P\,m}=\frac{M_{x}-F_{y}\,z_{0}}{F_{z}}.$$

*M_x_* and *F_y_* are the measured moment and force on the force plate. *z_0_* is the distance between the top plate surface and the true origin of the force plate.

## 6. Results and discussions

### 6.1. Mechanical design evaluation

**FIGURE 6:**
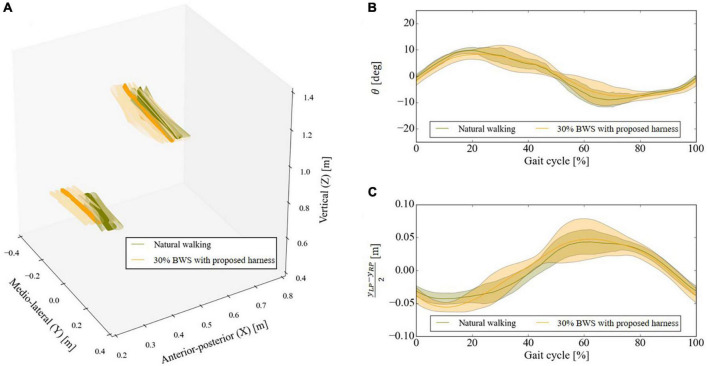
Results of motion capture experiment. **(A)** Three-dimensional displacement of vectors connecting the left and right acromie and posterior superior iliac spine (PSIS), **(B)** three-dimensional angle between two vectors that connect the left and right acromie and PSISs [see Eq. (7) and Figure 4B], and **(C)** center of the left and right PSIS position of the same representative participant. Solid lines are means calculated for all gait cycles, while the shaded regions represent ± 1 standard deviation from the mean.

Figure 6A shows the three-dimensional displacement of vectors connecting the left and right acromie and PSIS of Participant B (71-year-old female) in two conditions; natural walking or walking under laterally identical force-controlled 30% BWS condition wearing the proposed harness. The minimum detected number of gait cycles across conditions was 29. The origin of the mediolateral (Y) direction is at the center of the left and right PSIS positions of the participant. Figures 6B, C show the angle θ and the lateral displacement of the center position of the left and right PSIS of Participant B in two conditions. The trend within each gait cycle is qualitatively similar between the conditions. The root mean square error (RMSE) between the reference and measured forces, the average θs between natural and 30% BWS walking, and the average $\frac{y_{L\,P}-y_{R\,P}}{2}$ were evaluated (Table 2). While Plooij et al. (2021) argued that a higher support point impedes the forward-leaning of the upper body to take a step, and the mechanical structure inhibits the pelvis motion, the proposed harness poses negligible influence.

**TABLE 2:** Root mean squared errors in Section “5.1. Mechanical design evaluation.”

Participant	θ [deg]	$\frac{y_{L\,P}-y_{R\,P}}{2}$ [m]	*F*_*rL*_ + *F*_*rR*_ [N]
A	2.42	0.027	19.65
B	6.93	0.019	27.32
C	6.49	0.033	17.33

The above results suggest that the proposed harness has no significant effect on walking motion, and the trend of upper body motion is qualitatively equivalent to that of natural walking among three participants in the experimental setup of the current study. However, Plooij et al. (2021) compared propelling velocity difference; thus, additional evaluation may be needed in our study because the velocity could not be measured in the current experimental setup. Moreover, Van Thuc and Yamamoto (2016) and Tran et al. (2020) identified the difference in CoM/CoP displacement between the different amounts of unloading conditions due to the pendulum effect (the trunk of participants swung) at higher level BWS. Thus, the current evaluation is limited, and verifying the motion restriction due to the proposed harness with different BWS levels is necessary. In addition, Kwak et al. (2022) confirmed that under an impedance-controlled BWS walking condition, the RMSE of pelvic roll rotational angle was smaller than the BWS walking condition without impedance control. Although it has not been confirmed in our experiments, split-force-controlled walking may alter the influence on the upper body and pelvic motion. However, these evaluations are extended focuses for the current study and must be conducted with hemiparetic patients, prioritizing their safety. Thus, hereafter, we use a mass instead of involving human participants for force control evaluation.

### 6.2. Force tracking task with mass

**FIGURE 7:**
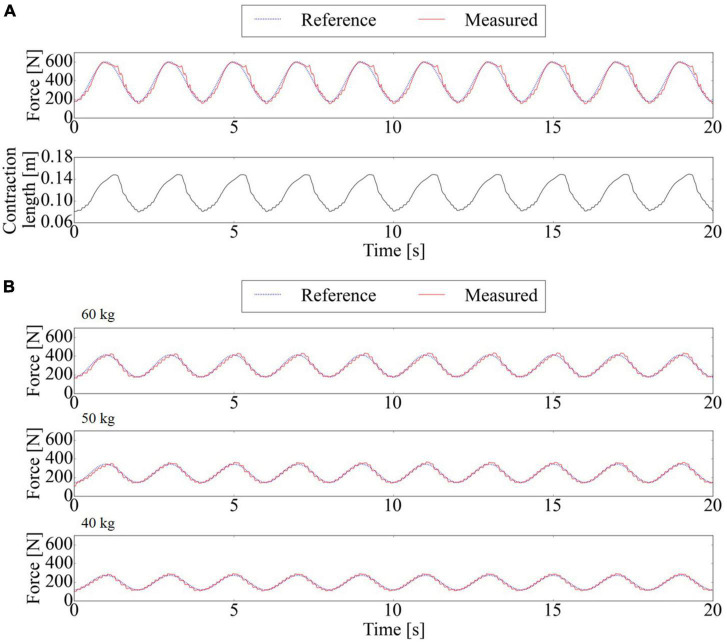
Force tracking performance of SF-BWS. **(A)** Reference and measured forces and the contraction length of PAM. PAM is controlled to maintain minimum 176.4 N support with the 211.8 N amplitude sinusoidal cyclic assist. Root Mean Squared Error (RMSE) was “29.84” N. **(B)** Force tracking performance using three different weights of masses with supporting rate per mass sinusoidally changed from 30% to 70%.

Split force-controlled-BWS with the force controller consisting of the feedback PAM model and PID feedback successfully tracks the reference force. Figure 7A top panel shows the force-tracking performance of SF-BWS. RMSE between the reference and measured force was 29.84 N within 20 s. Figure 7A bottom panel shows the PAM’s contraction length. The mass is in contact with the ground; thus, the change in the PAM contraction length was caused by the stretch of the string connecting the PAM and the mass. The range of operation was 6.91 cm. Note that the length is not controlled; thus, the PAM is extended when the reference force is smaller than the mass weight. In contrast, the weight is fully compensated when the PAM exerts 600 N force, and the length of the PAM becomes shorter and the contraction stops at the equilibrium point. Consequently, the length of the PAM is determined by the weight of the mass in this experiment. This experimental result shows that the force can be freely controlled in the range from 176.4 N to 600 N when the connection position of the mass is constant. This connection position can be arbitrarily changed according to the user’s waist height by adjusting the length of the climbing ropes and strings. In other words, by adjusting the length of the climbing rope and string connecting to the proposed harness, a large force can be generated, even when the PAM contraction length is short.

SF-BWS with the force controller successfully tracks the reference force with different masses (Figure 7B). RMSE between the reference and measured forces was 17.14 N at 60 kg mass, 12.88 N at 50 kg mass, and 10.69 N at 40 kg mass within 20 s.

### 6.3. Split-force control task with test bench

**FIGURE 8:**
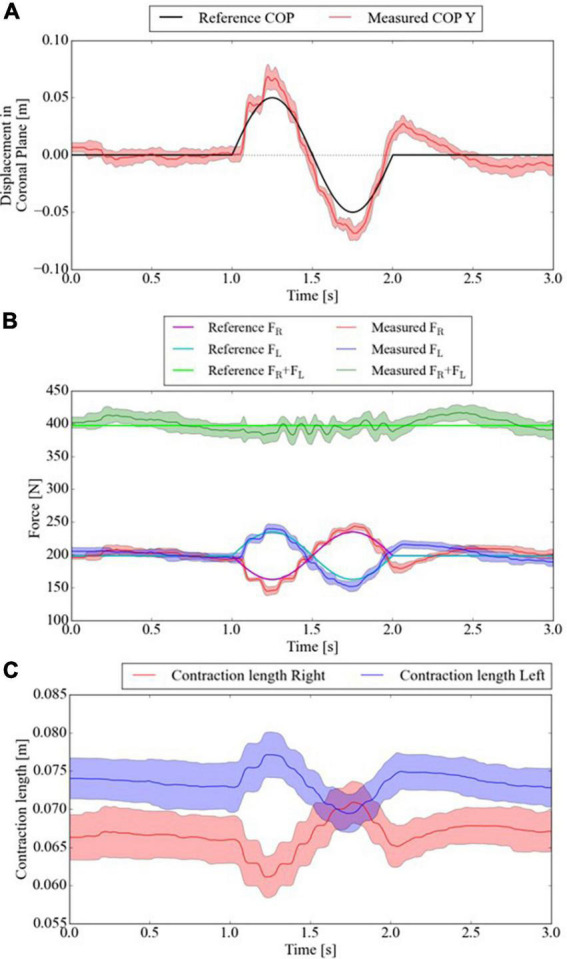
CoP of test bench tracks 1) 1-s single-sinusoidal reference. Mean and standard deviation of measured **(A)** CoP displacement in the Y-direction, **(B)** PAMs’ forces, and **(C)** contraction length within ten trials.

The reliability of controlling PAMs to oscillate the CoP under 60% BWS was sufficient, as more than 60% BWS was required to modulate spatiotemporal walking patterns in previous studies (Dragunas and Gordon, 2016; Tran et al., 2020). For the first experimental condition, the SF-BWS successfully accomplished CoP oscillation in a 1-s single-wave reference. Figure 8A shows the reference and the measured CoP position in the y-direction. The mean of RMSE between the reference and measured CoPs by the reaction force plate using Eq. (16) was 0.011 m for ten trials. Figure 8B shows the force tracking results. The RMSE of reference and measured force in the right PAM was 9.7 N, and that of the left PAM was 10.3 N within ten trials. Since the test bench did not move, the SF-BWS force control performance and the resulting CoP tracking were highly reproducible in the current study. The average of the measured total body weight support force (*F_L_ + F_R_*) was 399.2 N, while the total body weight support force is supposed to be 397.2 N.

The bench was never lifted off the reaction force plate or actively moved. The change in the PAM contraction length (Figure 8C) was caused by the stretch of the string connecting the PAM and the bench. The string length causes the difference between the left and right PAM contraction lengths because the string length is adjusted manually using the rope ratchets. The contraction length critically affects the force generated by the PAMs. Thus, better control of the initial setting of the string length is needed for more precise force control. In addition, the sinusoidal wave frequency is higher than that of the force-tracking task in Figure 7; thus, the delay caused by the air exhaust was more striking. Although the delay and the force errors can be compensated by adding the velocity terms of pressure and contraction rates and selecting a string that never stretches, the actual interaction with humans while walking increases the error. Moreover, PAM responsiveness for the desired force affects the user’s comfort (the time constant is added and set to 0.3 in our clinical trial for the patient’s comfort). Thus, further investigation of patient response to the intervention force with close collaboration with the clinical site is the future direction of SF-BWS development. The CoP acceptably tracked the reference in the mediolateral direction using the test bench.

**FIGURE 9:**
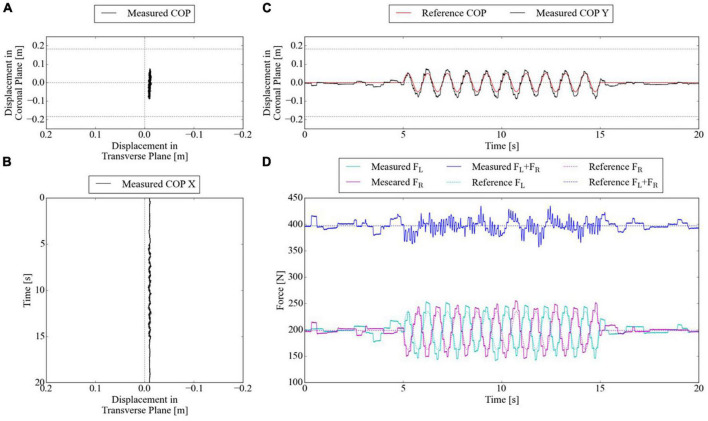
CoP of test bench tracks 2) 20-s block-designed sinusoidal reference. **(A)** CoP displacement in the horizontal plane measured by reaction force plate. **(B)** CoP displacement in X-direction in time series. **(C)** CoP displacement in Y-direction in time series. **(D)** PAMs’ forces in time series.

For the second experimental condition, based on the successful results of tracking the COP in Figure 8, we also attempted advanced tracking under conditions closer to the clinical conditions used in our previous conference paper. SF-BWS successfully achieved CoP oscillation in a 20 s block-designed reference. Figure 9A shows the CoP displacement measured using the reaction force plate. Since SF-BWS applies the forces in the coronal plane, the CoP displacement in the X-direction is negligible (it is also obvious from Figure 9B). The reference and measured CoP positions in the Y-direction are shown in Figure 9C. The RMSE between the reference and measured CoPs obtained using the reaction force plate [Eq. (16)] was 0.014 m within 20 s. The RMSEs were below 30% compared with the amplitude (0.050 m). Thus, the residual mean displacement of the mass CoP was sufficiently small compared to the reference range. The bench never actively moved, nor was it lifted off the reaction force plate. Thus, the robot was able to lead the shift in users’ body weight.

Figure 9D shows the force tracking results. RMSE of reference and measured force in the right PAM was 8.6 N, and that of the left PAM was 9.6 N within 20 s. The total body weight support force was assumed to be 397.2 N, and the average of the measured total body weight support force (*F_L_ + F_R_*) was 397.5 N. Thus, the developed SF-BWS was capable of assisting both pelvic rotations and constant body weight support.

## 7. Conclusion and future work

In this study, we introduced and evaluated the mechanical and control design of our newly developed SF-BWS robot. The robot can actively deliver large but compliant forces of two PAMs, which are independently transmitted via cables and uniquely shaped harnesses without rigid structures that impede walking movement. The upper body motion was comparable to natural walking. The SF-BWS was able to control PAMs in a force-tracking task using different simulated load weight setups and a split-force control task using a test bench. Precise force control of the two PAMs is necessary to perform these target tasks. The successful performance suggests that the SF-BWS has key functions in performing both pelvic rotations to assist with walking pattern modulation and supporting partial body weight for safe walking practice. Moreover, dual roles were achieved with a single robotic module. SF-BWS has already been applied in stroke patients, and interaction data are currently being collected. The pattern modulation performance of patients with hemiparetic stroke as well as the NcPAM benefits will be evaluated in future studies.

To extend the capabilities of the proposed SF-BWS robot, we will adopt an assisting strategy to support the smooth and natural walking movements of patients during gait training with physical therapists (Yagura et al., 2006). The physical therapist assists by manipulating the swing leg such that it lands heel first. Then, the therapist gently pushes the patient’s pelvis and transfers the body weight to the stance leg. The weight shift to the stance leg must be associated temporally with stepping movements (Dietz et al., 2002). As such, since pelvic motion depends on walking speed and step rate, BWS interventions should be adapted to human gait (Aoyagi et al., 2007; Badea et al., 2021). To support these types of movements in clinical situations, we are currently applying the developed SF-BWS to patients in a rehabilitation hospital (results are briefly reported in our conference abstract, Fujimoto et al., 2019). Our future research will focus on designing an optimal framework to derive CoP references for individual patients.

## Data availability statement

The raw data supporting the conclusions of this article will be made available by the authors, without undue reservation.

## Ethics statement

The studies involving human participants were reviewed and approved by the Ethics Committee of Advanced Telecommunications Research Institute International. The participants provided their written informed consent to participate in this study.

## Author contributions

AT, TT, KI, TN, and JM contributed to the study and experimental design. JM and IM contributed to project supervision. AT performed data acquisition and analysis. AT, TT, and KI prepared the manuscript. AT, TT, TN, HF, MH, NF, AJ, YH, IM, and JM participated in interpreting the results. All authors have read and approved the final manuscript.
